# Hybrid Refractive-Diffractive Lens with Reduced Chromatic and Geometric Aberrations and Learned Image Reconstruction

**DOI:** 10.3390/s23010415

**Published:** 2022-12-30

**Authors:** Viktoria V. Evdokimova, Vladimir V. Podlipnov, Nikolay A. Ivliev, Maxim V. Petrov, Sofia V. Ganchevskaya, Vladimir A. Fursov, Yuri Yu. Yuzifovich, Sergey O. Stepanenko, Nikolay L. Kazanskiy, Artem V. Nikonorov, Roman V. Skidanov

**Affiliations:** 1 Image Processing Systems Institute of the RAS—Branch of the FSRC “Crystallography and Photonics” RAS, Molodogvardeiskaya St. 151, Samara 443001, Russia; 2 Samara National Research University, Moskovskoye Shosse 34, Samara 443086, Russia

**Keywords:** diffractive-refractive hybrid optics, computational imaging, deep learning, lens optimization, image reconstruction

## Abstract

In this paper, we present a hybrid refractive-diffractive lens that, when paired with a deep neural network-based image reconstruction, produces high-quality, real-world images with minimal artifacts, reaching a PSNR of 28 dB on the test set. Our diffractive element compensates for the off-axis aberrations of a single refractive element and has reduced chromatic aberrations across the visible light spectrum. We also describe our training set augmentation and novel quality criteria called “false edge level” (FEL), which validates that the neural network produces visually appealing images without artifacts under a wide range of ISO and exposure settings. Our quality criteria (FEL) enabled us to include real scene images without a corresponding ground truth in the training process.

## 1. Introduction

The joint use of diffractive and refractive elements in optical imaging systems was first proposed in work [1], where diffractive optical elements were used to design a varifocal lens. However, the proposed design worked well only for monochromatic light. When used with white light, all shortcomings of the diffractive optics became apparent, producing strong image degradations due to heavy chromatic aberrations. It took 18 years until the next work [2] was published, which became foundational in the use of diffractive elements as chromatic aberration correctors. Since then, there has been an increase in the number of publications dedicated to this topic. In paper [3], it was shown that in addition to compensating for chromatic aberration, diffractive lenses were capable of compensating for spherical aberrations as well. In Ref. [4], industrially manufactured hybrid diffractive achromats were presented by Eastman Kodak Company. Unfortunately, another shortcoming of diffractive lenses, high-level light scattering, has prevented the wide use of refractive-diffractive systems in imaging optics, prompting continuing research efforts to address the said shortcoming [5,6,7]. Suggested solutions [5,6,7] require the manufacturing of 2-to-3-layer diffractive structures on spherical surfaces, which requires a more complex manufacturing process immensely to produce this refractive-diffractive lens, rarely available even in advanced labs. A diffractive lens on a flat substrate, on the other hand, can be produced with widely available fabrication equipment, enabling a wide range of applications. For instance, in Ref. [8], the diffractive lenses were used for a composite imaging system identical to a facet insect eye. In Ref. [9], the refractive-diffractive elements were used as a component of an artificial eye. In [10], a hybrid system was also used to increase the focal depth. The diffractive structure was constructed as a binary lens simultaneously exhibiting both light converging and diverging properties. As a result, two spatially separated on-axis foci were formed, between which images of near-same sharpness could be formed, thus eliminating the need for the focal length adjustment of this artificial lens. 

A significant number of publications were dedicated to the analysis of refractive-diffractive lenses [11,12,13]. A reflecting lens that reflects light inside the glass and has an annular aperture, with a diffractive element being the only element used to compensate for chromatic aberration, was analyzed in the paper [14]. While the idea proposed in paper [1] was extended and implemented at a new, higher-quality technology level in paper [15], the lens still exhibited the old shortcomings.

A combination of refractive-diffractive lenses with computational image reconstruction allowed us to reach the image quality that real-world applications demand. In previous works [16,17,18,19], deep learning-based image reconstruction was successfully used to compensate for the chromatic distortions typical in an optical system with a harmonic diffractive lens. Despite good results of the deep learning-based correction as measured by the peak signal-to-noise ratio (PSNR) on a test set at about 27 dB [16,20], reconstructed real scene images showed visible reconstruction artifacts. These artifacts were caused by the following features specific to real scenes as opposed to the training set: high dynamic range (HDR), camera gain, and lossy video compression. We modified our training procedure to overcome these reconstruction artifacts. 

In this work, we propose a hybrid refractive/diffractive camera lens based on the design proposed in work [3], where the capabilities of the diffractive optics to compensate for both chromatic and spherical aberrations was first demonstrated. Analyzing the performance of this lens, we show that the lens-aided aberration compensation creates an excellent point spread function (PSF) near the optical axis, with the off-axis PSF rapidly increasing with a growing angle and reaching unacceptable values at viewing angles as little as 6–8°. These off-axis aberrations play a key role in image degradation. In this work, we compensate for both chromatic and off-axis aberrations with a single diffractive lens designed specifically to perform the corrective function.

In order to address image degradations with image post-processing, deep learning-based methods can be used. To eliminate reconstruction artifacts for real scenes, we augmented the training set to better simulate the variety of the scenes. Our augmentation procedure includes two types of simulated image degradations: the camera gain (ISO) and the exposure shift. To measure the artifact levels, we designed our own criteria, which we called a false edge level (FEL), to select the best point of the model parameter space, resulting in artifact-free image reconstruction. We use a lighter version of U-Net [21] architecture which has a fast-training process with the best image reconstruction quality. 

The main contribution of this paper is two-fold.

(1) On the optical side, we describe our design process for the hybrid refractive-diffractive lens that minimizes chromatic and geometric aberrations from the concept to the manufactured prototype.
(2) On the software side, we present our deep-learning image reconstruction that combines a lab-captured dataset with real images extended with our image augmentation to obtain artifacts-free image reconstruction, with PSNR reaching 28 dB on test images and delivered a good visual quality for the captured real scenes.

## 2. Chromatic Aberration Compensation Design of the Diffractive Element of Our Refractive-Diffractive Optical System

**Figure 1:**
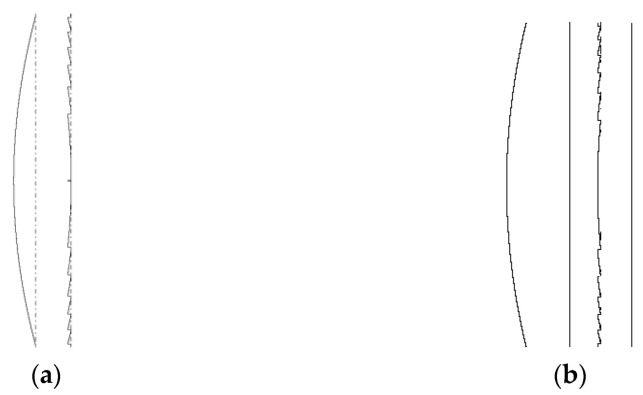
Images created with our inhouse-designed Harmony software of the doublet with refractive and diffractive elements to compensate for chromatic aberrations of two layouts: (**a**) a single lens with refractive and diffractive surfaces and (**b**) a system composed of a flat-convex lens and a diffractive lens on the surface of a flat substrate.

When incorporating diffractive optics into classical imaging lens systems, we can exploit the key difference between the two types of optics to yield the chromatic aberration correction effect: the refractive lens material’s dispersion and the dispersion of the diffractive lens focusing properties are opposite in sign. As the incident wavelength increases, the focal length of the refractive lens also increases, while the reverse effect occurs at the diffractive lens. With the properly selected parameters, a refractive/diffractive lens doublet (Figure 1) can cancel the chromatic aberration entirely for two incident wavelengths while reducing it significantly on the interval between them [2]. Although a single glass with refractive and diffractive sides is ideal (Figure 1a), this design is challenging to manufacture cost-effectively. Instead, in this work, we use the design shown in Figure 1b for the numeric modeling, manufacturing, image quality measurements, and for computational correction. Optical elements in our test setup are separated by a gap of 1mm, as illustrated in Figure 1b; the same distance is used in our analytical and numerical modeling.

Historically, this hybrid design was used for illustration purposes only because this combination of a standard spherical refractive lens and a standard diffractive lens approximating spherical element results in substantial geometric aberrations. Therefore, diffractive optics were incorporated into fairly complex optical systems [22,23] only to compensate for chromatic aberrations or for the combined chromatic/spherical aberrations [3]. However, the diffractive element is capable of more than just compensating for chromatic aberrations. Paper [24], where a diffractive element is incorporated into the optical design, is notable because they had to use a complex arbitrary-shape refractive element to compensate for geometric aberrations. Work [24] is an example of how the knowledge bias, in this case, expert knowledge of authors in refractive optics, can result in overlooking the rich capabilities of a well-designed diffractive element. With our experience in diffractive optics, we know that a single diffractive element is capable of approximating an arbitrary aspheric surface, however complex it is. In theory, because each individual diffraction zone can be treated as an optimization parameter, a diffractive element can be designed to compensate for both geometric and chromatic aberrations. 

Unfortunately, popular imaging optics design and modeling software, such as ZEMAX and CODE V, have poor functionality when it comes to the design of optical systems that include diffractive elements, let alone optimize their design. To design and optimize our novel systems with an arbitrary number of diffractive elements, we created our own software called HARMONY with a set of design and optimization tools that can be used for multiple refractive and diffractive elements using ray-tracing. We used HARMONY to design Earth imaging diffractive lens, launched to LEO last year [17]. More details about HARMONY can be found in [25].

Let us analyze the key formulae used when designing a refractive-diffractive achromatic doublet [9]. For the achromatization condition to be met, the focal lengths of a two-lens system at two different wavelengths need to be the same. For a refractive element, the focal length at the wavelength λ_1_ is given by the formula

(1) $$f\left(\lambda_{1}\right)=\frac{R_{1}R_{2}n\left(\lambda_{1}\right)}{\left[n\left(\lambda_{1}\right)-1\right]\left[n\left(\lambda_{1}\right)\left(R_{1}+R_{2}\right)-d\left(n\left(\lambda_{1}\right)-1\right)\right]},$$

where *R*_1_ and *R*_2_ are the radii of curvatures of the first and second surfaces, respectively, *d* is the lens thickness, and *n*(λ_1_) is the refractive index at the wavelength λ_1_. For a diffractive lens, the focal length is given by the formula

(2) $$f^{d}\left(\lambda_{1}\right)=\frac{\lambda_{0}f_{0}}{\lambda_{1}},$$

where λ_0_ is the operating wavelength, and *f*_0_ is the calculated focal length. The wavelength λ_2_ is derived using the same Equation (1):

(3) $$f\left(\lambda_{2}\right)=\frac{R_{1}R_{2}n\left(\lambda_{2}\right)}{\left[n\left(\lambda_{2}\right)-1\right]\left[n\left(\lambda_{2}\right)\left(R_{1}+R_{2}\right)-d\left(n\left(\lambda_{2}\right)-1\right)\right]}$$

(4) $$f^{d}\left(\lambda_{2}\right)=\frac{\lambda_{0}f_{0}}{\lambda_{2}}$$

The combined focal length of the system with refractive and diffractive elements is given by

(5) $$F=\frac{ff^{d}}{f+f^{d}-D}$$

where *D* is the distance between the refractive and diffractive lenses (1 mm in our design).

The condition for the combined focal length to be the same at two wavelengths is:

(6) $$\frac{f\left(\lambda_{1}\right)\lambda_{0}f_{0}}{\lambda_{1}\left[f\left(\lambda_{1}\right)+\frac{\lambda_{0}f_{0}}{\lambda_{1}}-D\right]}=\frac{f\left(\lambda_{2}\right)\lambda_{0}f_{0}}{\lambda_{2}\left[f\left(\lambda_{2}\right)+\frac{\lambda_{0}f_{0}}{\lambda_{2}}-D\right]}$$

A simple rearrangement yields:

(7) $$\lambda_{0}f_{0}=\frac{f\left(\lambda_{1}\right)f\left(\lambda_{2}\right)\left(\lambda_{1}-\lambda_{2}\right)-D\cdot \left[f\left(\lambda_{1}\right)\lambda_{2}-f\left(\lambda_{2}\right)\lambda_{1}\right]}{f\left(\lambda_{1}\right)-f\left(\lambda_{2}\right)}$$

Considering that the best result will be achieved if the calculated wavelength is in the middle between the wavelengths λ_1_ and λ_2_ $\lambda_{0}=\frac{\left(\lambda_{1}+\lambda_{2}\right)}{2}$ Equation (7) can be rearranged to

(8) $$f_{0}=\frac{2\cdot \left\{f\left(\lambda_{1}\right)f\left(\lambda_{2}\right)\left(\lambda_{1}-\lambda_{2}\right)-D\cdot \left[f\left(\lambda_{1}\right)\lambda_{2}-f\left(\lambda_{2}\right)\lambda_{1}\right]\right\}}{\left(\lambda_{1}+\lambda_{2}\right)\left[f\left(\lambda_{1}\right)-f\left(\lambda_{2}\right)\right]}$$

**Figure 2:**
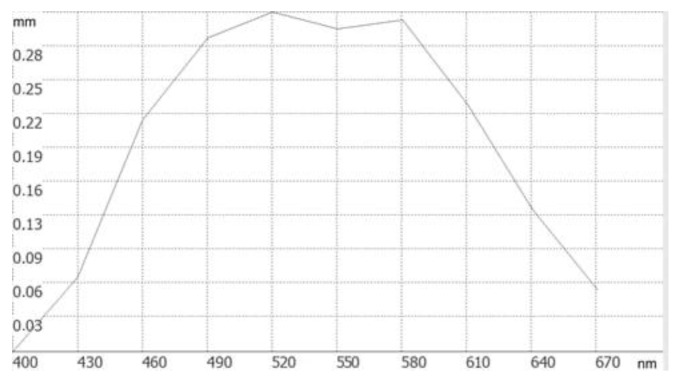
The focal plane shift vs. the wavelength for a refractive-diffractive lens doublet is calculated based on Equations (1)–(8).

Using our analytical solution, we calculated the parameters of our refractive-diffractive doublet and, using our HARMONY software, modeled the focal plane shift as the incident wavelength increases from 400 n through 670 nm with a step of 30 nm. As Figure 2 shows, an optical system is configured based on Equations (1)–(8) fails to produce an optimal result. Specifically, there is a non-zero shift at a second boundary wavelength of 670 nm.

**Figure 3:**
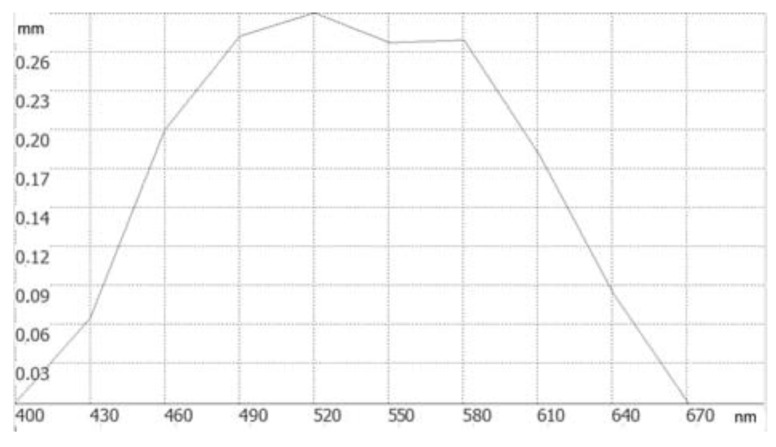
Focal plane shift vs. the wavelength for a refractive-diffractive doublet, optimized in the HARMONY software, with zero shift at boundary wavelengths.

We then performed precise optimizations of the chromatic aberrations in our HARMONY software and produced a different from the analytical solution (Figure 3). The relative difference between the analytically and numerically designed parameters was in the range of 5 to 7%. For instance, for a refractive flat-concave lens with a 75-mm focal length made of BK7 glass, the Equations (1)–(9) gives the *f*_0_ value of 647 mm, while HARMONY software suggests the right value of *f*_0_ is 626 mm. Figure 3 shows how the focal plane shifts with the incident wavelength for a system optimized with the HARMONY software.

With our HARMONY software, we also minimized the width of the off-axis PSF. Microrelief heights in each diffraction zone were treated as free parameters and individual coordinates, and the optimization was carried out using a coordinate descent solver.

As shown in Appendix A, our optimization successfully achieved the desired achromatization effect, resulting in a minimized point spread function (PSF). The intensity distribution was accurately measured and showed that our optimization significantly reduced the PSF width from 11.2 μm to 7.1 μm, a 36% decrease.

## 3. Manufacturing of the Diffractive Lens

To evaluate the imaging quality of the proposed hybrid lens, we constructed a prototype imaging system that combines both refractive and diffractive elements using the parameters computed in the previous section. The lens was designed for a principal wavelength of 535 nm, with a resulting optimal microrelief height of 1000 nm.

**Figure 4:**
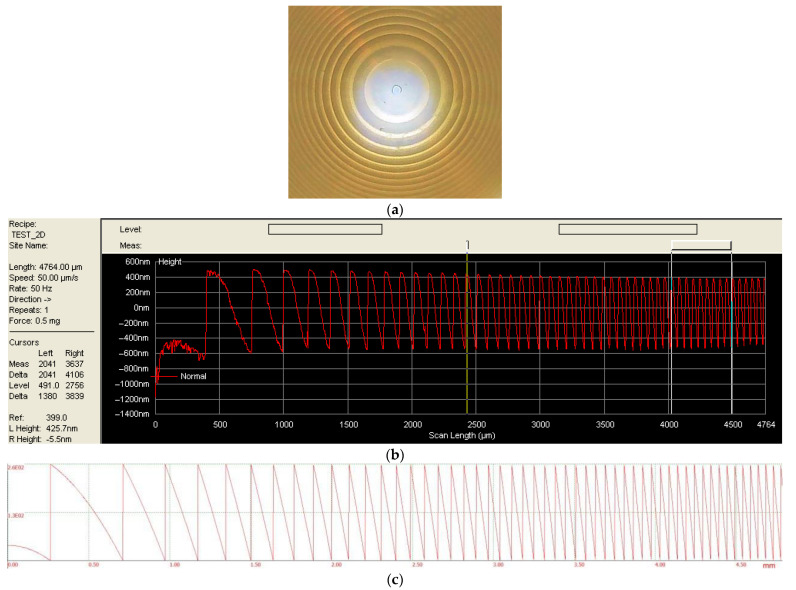
(**a**) Our reflecting harmonic lens with the annular microrelief, observed through the microscope, (**b**,**c**) observed and calculated cross-section microrelief profiles, respectively.

The diffractive lens was fabricated by the direct laser writing in a photoresist using a laser writing station CLWS-2014. The diffractive lens has a 626-mm focal length and a 10-mm diameter, as computed in the previous section. The focal length and the diameter were chosen to match the parameters of our refractive flat-convex optical element. The image of the central part of the manufactured lens is shown in Figure 4a with the lens radial cross-section depicted in Figure 4b. 

**Figure 5:**
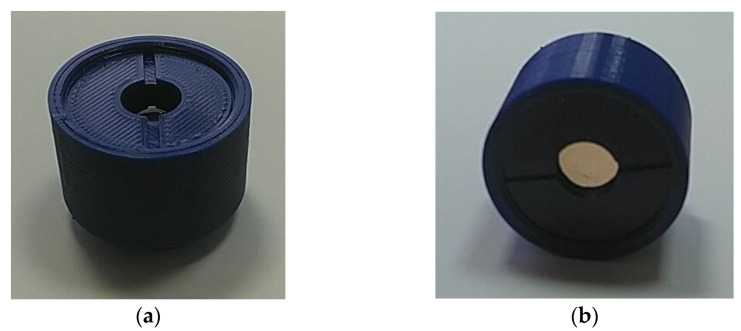
An exterior view of the refractive-diffractive doublet in the 3D-printed plastic casing: top view (**a**), side view (**b**).

As can be seen in Figure 4b, the measured microrelief height matches the designed target height (about 1000 nm). In order to house both optical elements, the lens doublet was placed in a 3D-printed plastic casing, printed with a resolution of 20 μm. An exterior view of the prototype camera lens is shown in Figure 5. The Basler acA1920-40uc USB 3.0 camera was attached to this lens assembly for the image capture and our reconstruction experiments.

## 4. Deep Learning-Based Image Reconstruction

### 4.1. Deep Learning-Based Image Reconstruction Overview

The image reconstruction that is effective for our diffractive optic-based imaging system is similar to a single image super-resolution (SISR) task. There are various deep-learning solutions that can produce visual-pleasing results with high PSNR and SSIM values for a SISR task [26,27]. Most of these methods are based on the known image degradation models and range from simple downsampling with a bicubic upsampling [28,29,30,31,32] to more recent works, relying on blurring kernel degradation [30,31,32]. When applied to real-world images, these algorithms suffer from artifacts because the real image degradation is usually too complex [33] or has a non-local behavior that depends on the image content [16,34]. Artifact-free results can still be achieved with techniques described in [33,35,36]. A meta-transfer learning-based training procedure [35] can make a network adaptive to a new degradation within a few iterations at the inference. Work [36] has flexible adaptation to degradations based on the learned representations. A higher-order degradation process that is based on simple degradations (such as blur, resize, noise, etc.) is proposed in [33] to model real-world degradations. A similar approach could be useful for modeling the degradations that are inherent to diffractive optics.

To build a semi-real dataset for supervised learning, we use a capture-from-screen laboratory setup [16] with a laptop connected to a UHD LCD monitor and Basler acA1920-40uc USB 3.0 camera with our doublet lens system. However, this setup has three main differences from real scene capturing: a higher dynamic range, camera gain, and lossy video compression. Unlike real-world scene capture, which can involve varying camera gain (ISO) and exposure, our setup with up to 200 lux of screen illumination produces images with consistent characteristics. When combined with lossy image compression, these image-specific parameters can cause reconstruction artifacts, as analyzed in works [16,17].

In this work, we propose a method for eliminating reconstruction artifacts by augmenting the training data with simulated image degradations that include variations in camera gain (ISO) and exposure shift. While lossy compression degradation was addressed in [16], in this work, we decided not to introduce compression degradation. Although our data augmentation helped to improve the quality, we decided to evaluate the level of reconstruction artifacts on real image patches during the training process to identify the best point in the parameter space. This approach resulted in the nearly complete elimination of reconstruction artifacts in the reconstructed real-world images.

To measure the artifact levels, we introduced the false edge level (FEL) criteria, which enabled us to incorporate real-world images into our training process. The FEL criteria are based on edge detection in real-world images and do not require a ground truth image. In work [37], a method for evaluating the quality of detail restoration in video super-resolution was proposed, called edge restoration quality assessment (ERQA), which was also based on edge detection. While this work showed that edge restoration is critical for human perception of detail restoration, their method required ground truth images. In our approach, we can use the edge-based estimation of reconstruction artifacts without ground-truth information.

As demonstrated in previous works [20,34], diffractive optics can cause two types of image degradation: local degradation, which is caused by chromatic aberration, and non-local, content-aware chromatic shift, which is caused by the redistribution of energy between the secondary diffractive orders of the lens. Since these degradations affect areas larger than 200 pixels in width in our setup [34], we use a CNN with a receptive field wider than 200 pixels, which is based on the modified U-Net [21] architecture. U-Net-based architectures were successfully used for image reconstruction in diffractive optic-based imaging systems before [16,17,18,19].

### 4.2. False Edge Level (FEL) Criteria

**Figure 6:**
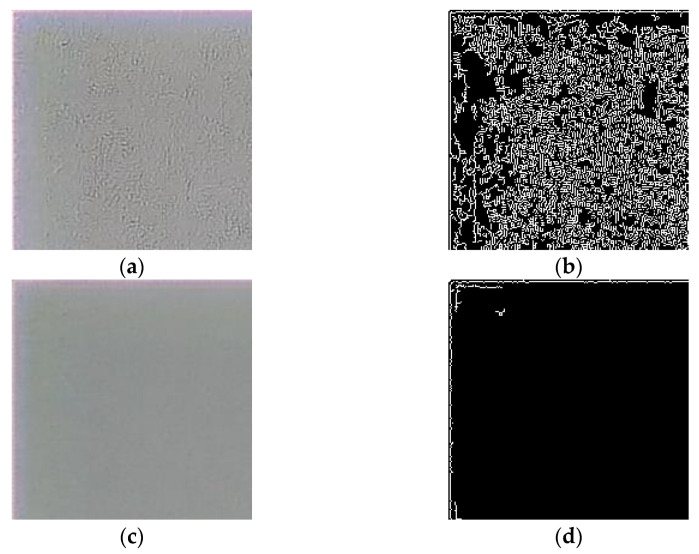
Edge detection examples: (**a**,**c**) reconstructed patches of real-world images with FEL of 30.69% and 1.28%, respectively; (**b**,**d**) results of the Canny algorithm of (**a**,**c**), respectively.

Observing the neural network performance on the real-world images, we noticed that reconstruction artifacts look resemble contours (Figure 6a,b). To assess the level of artifacts, we calculate the percentage of contour pixels in a patch that should not contain contours. We called this metric “false edge level” (FEL), defined as follows:

(9) $$\mathrm{FEL}=\frac{1}{NM}\underset{\begin{array}{c} 0\leq i\leq N-1\\ 0\leq j\leq M-1 \end{array}}{\sum }E_{ij}\cdot 100,$$

where *E* is *a* N×M binary edge map produced by the Canny algorithm with thresholds set to 0 and 70, E has to be normalized to [0,1] before calculating the FEL. The thresholds for the Canny algorithm were experimentally selected to find the optimal match between visual artifact levels in real-world images, and the percentage of contour pixels after edge detection was performed. Figure 6 shows examples of the edges detected by the Canny algorithm for real-world image patches and the corresponding FEL values. Our choice of the Canny algorithm was inspired by the work [38], where the Canny algorithm was successfully used for the ERQA metric calculation. 

### 4.3. Dataset Capture and Data Augmentation Strategy

We collected our dataset using a capture-from-screen laboratory setup successfully used before, as described in [16,17,20,34]. In this setup, a laptop is connected to the UHD LCD monitor with an IPS panel and 163 ppi resolution via an HDMI cable, where this monitor serves as an image generation device, and a Basler acA1920-40uc USB 3.0 camera, which serves as an image capturing device. The software which we developed automates the process where images are displayed by the monitor and then captured with customizable timing. A calibration image with markers helps to match captured images with the original ones. The training, test, and validation sets contained 1244, 613, and 21 pairs of the input and the ground truth 1024*1024 RGB images, respectively. The training on the display-captured images results in the network with a high level of mean PSNR value and good visual quality on the test set. However, when applied to real-world images, a CNN-based reconstruction produced undesirable artifacts [16,17].

Since lossy image compression in this work was not used, we considered two types of degradations: camera gain noise (ISO noise) and exposure change. We propose to augment the training dataset by modeling these degradations. To model ISO noise, we used Poissonian-Gaussian noise [39,40], where a Poissonian component models the photon sensing and a Gaussian component for the remaining stationary disturbances in the output data. We applied Poissonian-Gaussian noise to input images to simulate camera ISO noise with a probability of 0.5. We used the algorithm implemented in the Albumentations library [41]. The intensity parameter of the ISO noise modeling algorithm was randomly selected from {0.1, 0.2, 0.3}.

To adjust the image exposure, we employed a low-light image enhancement algorithm, as described in [42]. The algorithm is based on a camera response model that relates the irradiance of the camera sensor to the pixel values in the image. The algorithm for enhancing images is based on estimating a camera response model using the histogram characteristics of two images with different exposure settings and an exposure ratio map. This allows us to adjust the exposure of the image without introducing color and lighting distortions. We apply this exposure adjustment after adding ISO noise to the image with a probability of 0.1.

### 4.4. Network Architecture

We use a modification of the U-Net architecture [26], which was successfully applied for post-processing images captured by harmonic diffractive lenses [27,28]. The original U-Net architecture as follows: 

C64-C128-C256-C512-C512-C512-C512-C512 (Encoder).

CD512-CD512-CD512-C512-C256-C128-C64-C3 (Decoder), C3.

Each encoder block Ck has a convolutional layer, batch normalization, and a ReLU activation function, where the number of filters is denoted by *k*. A dropout layer is added before each activation layer in decoder blocks. The filter size is 4 × 4 pixels. The architecture has skip connections between each layer *i* in the encoder and layer *(n-i)* in the decoder, where *n* is the total number of layers. The last layer has a tanh activation function. 

In our work, we implemented a lighter architecture:

C64-C128-C256-C512-C512 (Encoder), CD512-C256-C128-C64 (Decoder), C3.   (10).

Although this lighter version of the network is faster to train because it has three times fewer trainable parameters and requires less memory, its image reconstruction quality is comparable to that of a more computationally expensive architecture, as measured by the mean peak signal-to-noise ratio (PSNR).

### 4.5. Training with the FEL Criteria for the Artifact-Free Reconstruction

For our training, we chose an ADAM optimizer [38] with $\beta_{1}=0.5$ and $\beta_{2}=0.999$ and a learning rate of 0.0002. In all our experiments, we used an $l_{2}$ loss function. In previous works [16,17], the best point of the parameter space was selected by a mean PSNR value calculated on the validation set. In our work, we use our FEL criteria on a real-world image patch to find the best parameters to minimize reconstruction artifacts.

**Figure 7:**
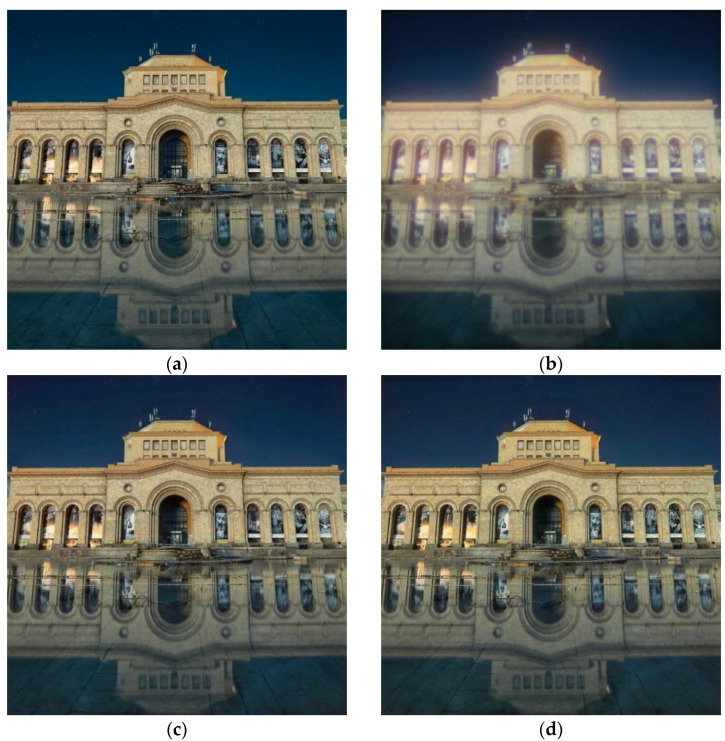
An image reconstruction example: (**a**) the ground truth image; (**b**) the image captured with our hybrid lens without post-processing (18.57 dB PSNR); (**c**,**d**) reconstructed images with the best point selected using max-PSNR (28.03 dB) and min-FEL criteria (26.86 dB), respectively.

We trained the CNN (10) on the display-captured train set for 200 epochs. The meaning of PSNR and FEL were calculated after each epoch. We selected two points corresponding to the maximum mean PSNR on the validation set (max-PSNR criteria) and the minimum FEL (min-FEL criteria) on the 200×200 patch of a real-world image. We feed the test set to both models initialized by two selected parameter points. Figure 7 shows an example of a test image reconstructed by these models. As can be seen in Figure 7, both reconstructed images (Figure 7c,d) are almost visually identical to the ground truth image (Figure 7a): the results for the max-PSNR criteria results in 1.17 dB-higher PSNR value than does the min-FEL, as measured using the test image.

**Figure 8:**
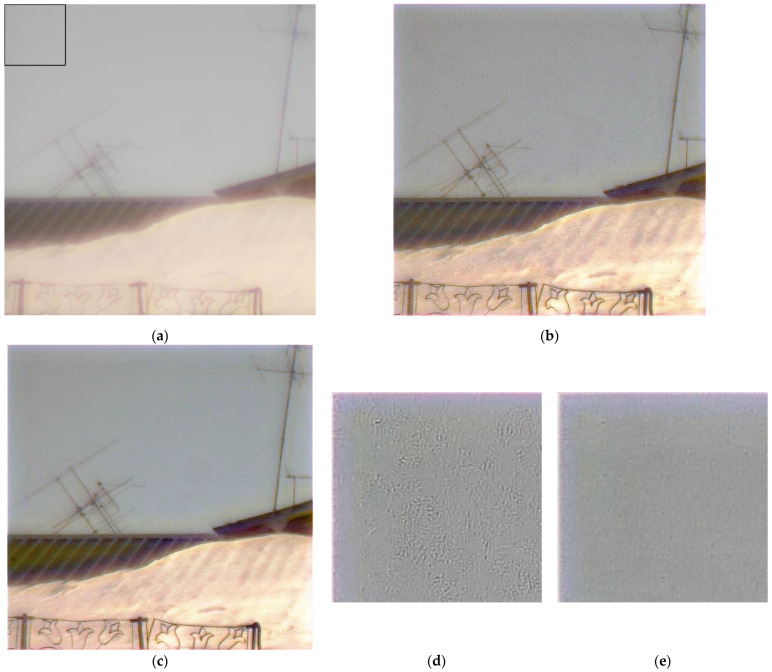
An example of real-world image reconstruction: (**a**) image captured by our hybrid lens with a 200 × 200 patch highlighted by the black rectangle; (**b**,**c**) reconstructed images using the best point selected by the max-PSNR and by the min-FEL criteria, respectively; (**d**,**e**) patches corresponding to the max-PSNR (FEL is 30.69%) and min-FEL criteria (FEL is 14.48%).

An example of real-world image reconstruction is shown in Figure 8, where the black rectangle in the left upper corner highlights the patch we used for the FEL calculation (Figure 8a). In Figure 8b,c, we show reconstructed images with the best point selected with the max-PSNR and min-FEL criteria, respectively. Patches of CNN-reconstructed images (Figure 8d,e) show that the min-FEL and max-PSNR, when used as optimization criteria, produce visually different results, where a cleaner image (Figure 8e) has a 16.21% lower FEL, confirming that min-FEL results in higher quality, as perceived by a human, images.

### 4.6. Data Augmentation Experiments

**Figure 9:**
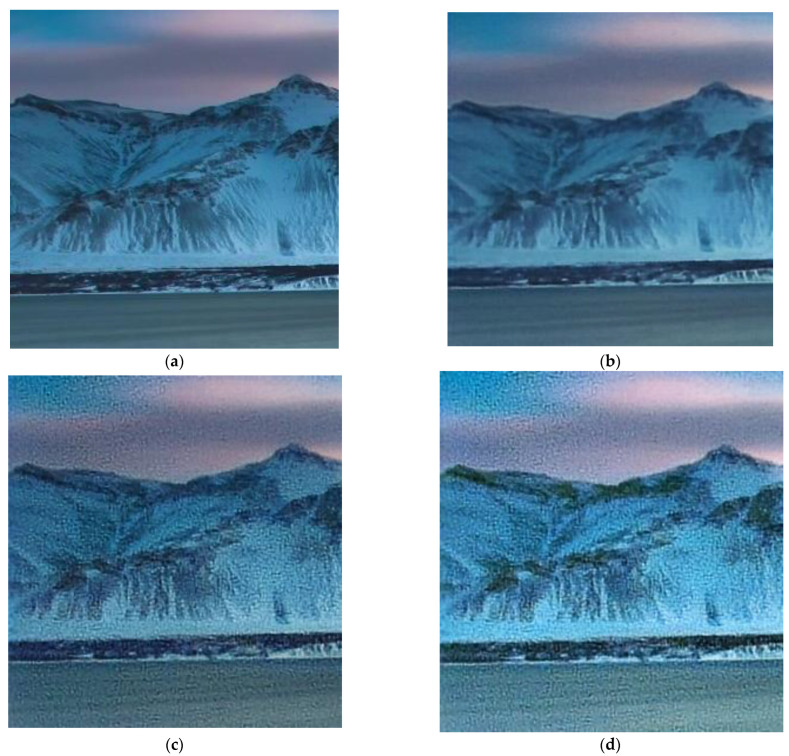
Artifacts of the CNN reconstruction of a test image: (**a**) a ground truth image; (**b**,**c**), and (**d**) CNN reconstruction of the captured image without any preprocessing, with ISO noise added, and with both ISO noise added and increased exposure, respectively.

**Figure 10:**
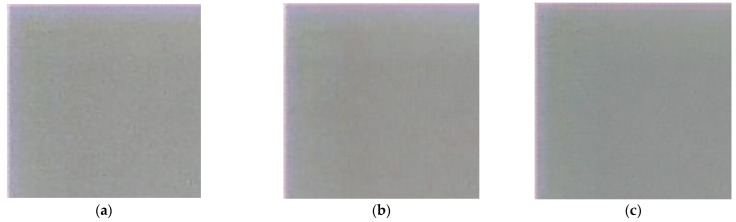
Reconstructed real-world image patches for different data augmentation from Table 1: (**a**) ISO noise with a probability of 0.5 and intensity of 0.1; (**b**) ISO noise with a probability of 0.5, intensity randomly selected from {0.1; 0.2; 0.3}; (**c**) ISO noise with a probability of 0.5, intensity randomly selected from {0.1; 0.2; 0.3}, and the adjusted exposure.

The inspiration for our data augmentation came from an observation that the reconstruction artifacts, which we saw with real-world images (Figure 8b–e), are visually similar to the artifacts generated by the reconstruction of the monitor-generated images with artificially added camera gain and exposure changes. Figure 9 shows how a test image (Figure 10a) looks after being captured by our test setup and CNN-reconstructed without adding any degradations (Figure 10b) and with added ISO noise between the reconstruction (Figure 9c) and with both ISO noise and exposure increase added (Figure 9d). The artifacts we observe look similar to those visible in the reconstructed real-world images (Figure 7b,d). 

With this observation in mind, we first augmented the training dataset with an ISO noise modeling algorithm with a probability of 0.5 and the intensity randomly selected from {0.1, 0.2, 0.3}. Then we shifted image exposure with a probability of 0.1. Reconstruction results for different train image augmentation parameters are demonstrated in Table 1.

**Figure 11:**
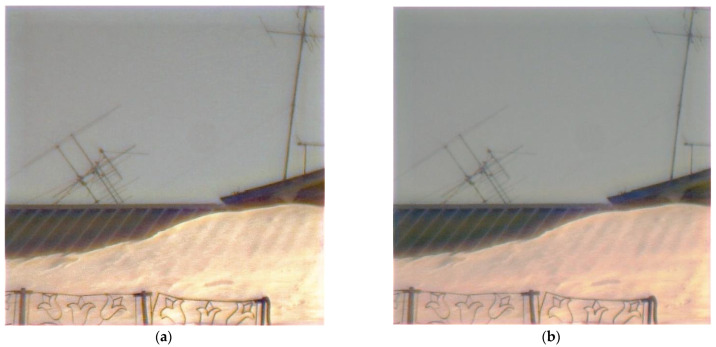
Reconstruction of the real-world image (Figure 8a) by the CNN trained on the augmented training set by (**a**) adding ISO noise with different intensities (Table 1, row 3); (**b**) by adding both ISO noise and exposure shift (Table 1, row 4).

Table 1 shows that the highest PSNR value is achieved when we use ISO noise augmentation with the intensity of 0.1 (row 2 in Table 1), while the minimum FEL is achieved with a higher augmentation variability, including ISO noise with three levels of intensity and exposure change (row 4 in Table 1). We already know that a smaller FEL corresponds to the visually better reconstruction quality of real-world images (Figure 8c and Figure 10). However, the PSNR metric on the test set suffers the more diverse the augmentation we apply. Figure 10 demonstrates reconstructed patches of a real-world image (Figure 8a) for the min-FEL criteria. Figure 10b has almost the same FEL value (just 0.06 lower) as Figure 10c, and the two are not surprisingly subjectively similar. After we visually compared the full-sized reconstruction images (Figure 11), we chose the augmentation, which includes applied ISO noise and exposure shift (Figure 11b).

Table 1 shows that the highest PSNR value is achieved when we use ISO noise augmentation with the intensity of 0.1 (row 2 in Table 1), while the minimum FEL is achieved with a higher augmentation variability, including ISO noise with three levels of intensity and exposure change (row 4 in Table 1). We already know that a smaller FEL corresponds to the visually better reconstruction quality of real-world images (Figure 8c and Figure 10). However, the PSNR metric on the test set suffers the more diverse the augmentation we apply. Figure 10 demonstrates reconstructed patches of a real-world image (Figure 8a) for the min-FEL criteria. Figure 10b has almost the same FEL value (just 0.06 lower) as fFigure 10c, and the two are not surprisingly subjectively similar. After we visually compared the full-sized reconstruction images (Figure 11), we chose the augmentation, which includes applied ISO noise and exposure shift (Figure 11b).

**Figure 12:**
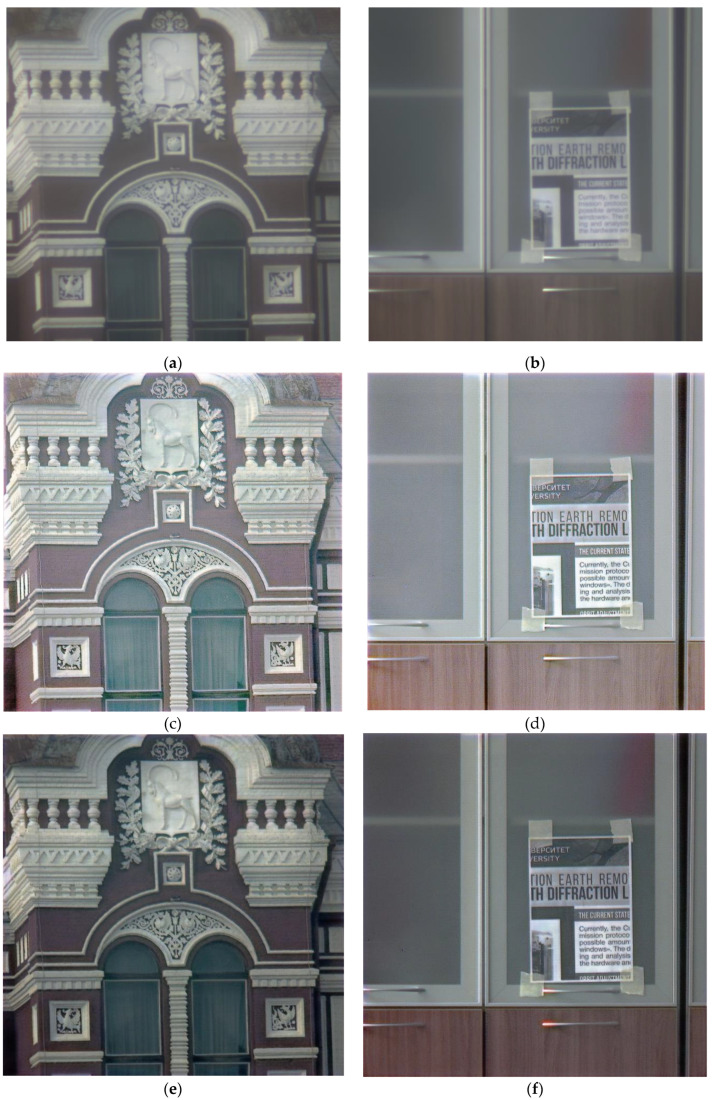

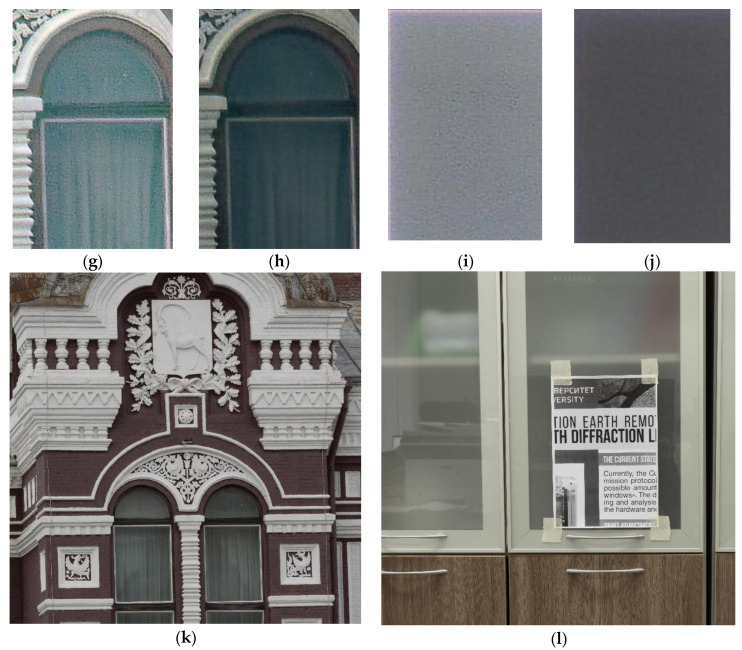
Reconstruction of the real-world images: (**a**,**b**) captured by the hybrid lens; (**c**,**d**) reconstructed with a CNN trained on the data with no augmentation; (**e**,**f**) reconstructed with a CNN trained on the augmented data; (**g**) a patch of (**c**); (**h**) a patch of (**e**); (**i**) a patch of (**d**); (**j**) a patch of (**f**); (**k**,**l**) captured by a standard refractive lens.

**Figure 13:**
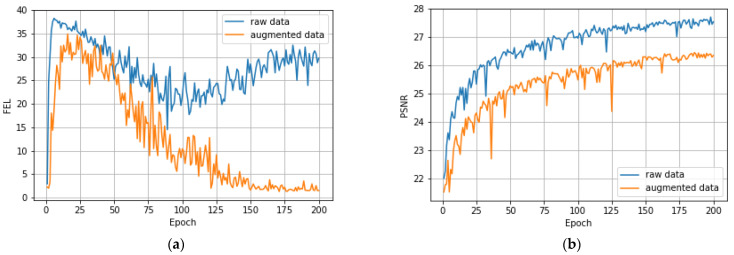
Quality metric comparison between the training on the acquired raw data with no augmentation (Table 1, Row 1) and on the augmented data with added ISO noise and exposure shift (Table 1, Row 4) (**a**) FEL calculated on the real-world image patch, (**b**) PSNR calculated on the validation set.

Figure 12 below shows other reconstruction examples of real-world images captured under different conditions. As can be seen in Figure 12, augmentation provides a noticeable improvement in visual quality and lowers the number of artifacts. Figure 13a shows the comparison of FEL values while training on the raw data without augmentation and on the augmented data. While training on the augmented data results in significant performance improvements in terms of the FEL, PSNR became somewhat lower with augmented data compared to the raw data (Figure 13b). Our use of FEL complements a more traditional PSNR measure to find an artifact-free image reconstruction solution.

### 4.7. Final Training Settings

For image reconstruction, we used a lightweight version of U-Net as described in Section 4.4. For datasets captured under different conditions, the light version of U-Net produced PSNR values on the validation set that fluctuated around PSNR values produced by the full U-Net. The convergence comparison, shown in Appendix B, confirms our choice of light architecture to find the best training settings in our research.

## 5. Conclusions

Our ambitious goal was to create a hybrid refractive/diffractive lens duplex, which, when combined with properly designed software post-processing, can produce visually high-quality images taken in the real world. To produce the results, we describe in this paper, we had to overcome a long list of challenges that were difficult individually and even more complex when combined in a hardware/software system with multiple interdependent components and produced a working system that performs well not only in the artificial environment of the lab but, more importantly, in the field. Specifically, we were able to accomplish the following (which we also describe in this paper in detail):

- We designed and optimized our hybrid lens system in the in-house software HARMONY to compensate for the lack of sufficiently powerful capabilities in widely available optical simulation tools. With full modeling flexibility, we designed the diffractive element to compensate for off-axis geometric aberrations of the refractive element and ensured that chromatic aberrations reached zero for two boundary wavelengths, ensuring robust performance on the whole visible spectrum. For the manufacturing, we used widely available laser writing hardware, which ensures the reproducibility of our results and allows for inexpensive mass production later.
- For image post-processing, we deployed an end-to-end deep learning-based image reconstruction with the architecture inspired by the UNet. To generate images used for training, we built a straightforward capture-from-screen automated laboratory setup. Intensive illumination ensured high-quality capture, and we artificially added ISO noise and exposure adjustments to augment the test set to ensure that our apparatus could perform well in a variety of lighting conditions outside of the capture setup.
- Initial experiments using a widely used PSNR metric for quality assessment showed that our neural network training produced inferior results when real-world pictures were processed. With a non-augmented test set of 613 images, we achieved a PSNR of 28.09 dB. When augmented with ISO noise and exposure adjustments, PSNR went down to 27.08 dB on the test set but showed better visual results with real-world images. Seeing the limitations of PSNR for our scenario, we invented a novel quality validation criterion that is aligned with human perception of quality, which we called FEL (false edge level) criteria. This allowed us to confirm that our trained neural network performs exceptionally well when it reconstructs real-world images often made under challenging lighting conditions. To argue our selection of this validation criterion, we present the data and images comparing the performance of the reconstruction with PSNR versus FEL, with FEL being a clear winner despite the fact that the resulting images have somewhat lower PSNR on the test set. The key to this validation advancement was not only the introduction of FEL but our use of a real image patch during the training without the need to produce a corresponding ground truth image.

In this paper, we describe the solutions we developed to overcome numerous challenges in building our hybrid refractive/diffractive camera setup, which can be the basis for the development of mass-produced, lightweight, high-quality hybrid imaging optics. Our min-FEL quality criterion can potentially be of greater importance for the broader field of image processing and will be thoroughly analyzed in subsequent research.

## Figures and Tables

**Table 1:** Comparison of metrics values for different augmentations.

№	Augmentation	The Best Point Selection Criteria	PSNR of Validation Set (dB)	FEL (%)	PSNR of the Test Set (dB)	Reconstructed Patch, Min-FEL Criterion
1	No augmentation	Max-PSNR	27.71	30.69	27.68	Figure 8d Figure 8e
		Min-FEL	26.56	14.48	27.01	
2	ISO noise with a probability of 0.5 and intensity of 0.1	Max-PSNR	27.52	3.32	28.09	- Figure 10a
		Min-FEL	27.13	2.83	27.65	
3	ISO noise (0.5 probability), random intensity 0.1, 0.2, 0.3	Max-PSNR	27.42	1.47	27.4	- Figure 10b and Figure 11a
		Min-FEL	27.29	1.35	27.37	
4	ISO noise with a probability of 0.5, random intensity 0.1, 0.2, 0.3; varying exposure	Max-PSNR	26.46	1.69	27.08	- Figure 10c and Figure 11b
		Min-FEL	26.2	1.29	26.91	

**Figure A1:**
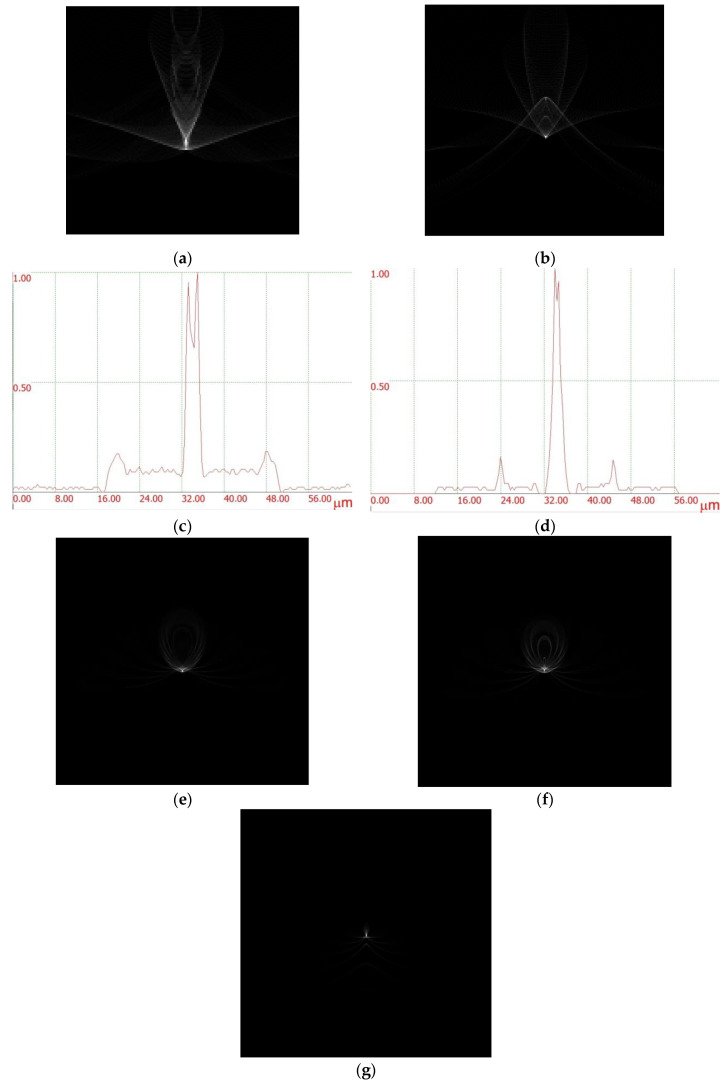
PSF at an 8° angle with the axis (**a**) before and (**b**) after the optimization within a 64*64 μm square; their corresponding horizontal sections are shown in (**c**,**d**); (**e**)—after the optimization for λ = 400 nm, (**f**) after the optimization for λ = 670 nm, (**g**) after the optimization for λ = 550 nm.

**Figure A2:**
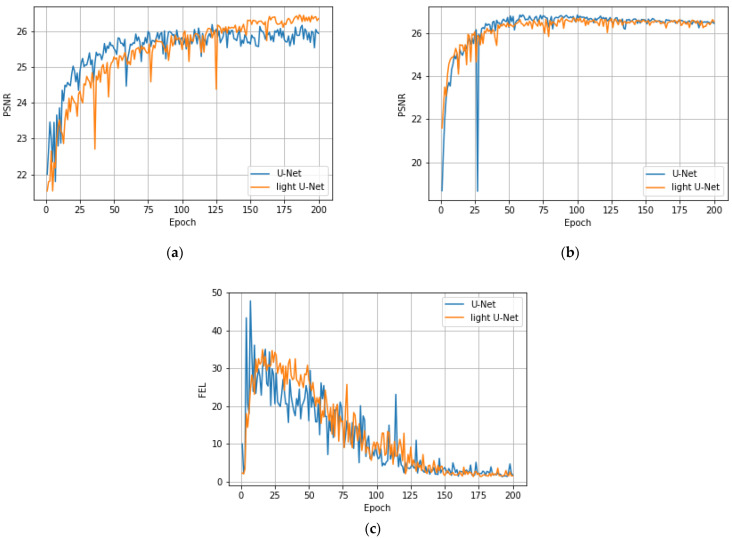
Comparing training convergence of a basic U-Net-like architecture with the light-weight architecture (10): (**a**,**b**) PSNR, calculated at each epoch on the validation set captured by the hybrid lens and by MDL, respectively; (**c**) FEL, calculated at each epoch on the real image patch captured by the hybrid lens.

## Data Availability

Not applicable.

## Appendix A. Estimation PSF of the Hybrid System

Figure A1 presents PSFs for our refractive-diffractive lens doublet, respectively, before and after the optimization, computed using our software. Figure A1e,g and Figure 4f show PSF at wavelengths of 400 nm, 670 nm, and 550 nm, respectively:

As shown in Figure A1, our optimization successfully achieved the desired achromatization effect, resulting in a minimized point spread function (PSF). The intensity distribution was accurately measured and showed that our optimization significantly reduced the PSF width from 11.2 μm to 7.1 μm, a 36% decrease.

## Appendix B. Comparing Training Convergence of the CNN-Based Image Reconstruction Models

Figure A2a shows the comparison of PSNR values while training for both architectures with the same hyperparameters using the dataset captured by the hybrid lens. Figure A2b shows the same experiment for a dataset captured by the MDL. However, for the FEL metric, Figure A2c shows the same result for both architectures. At the end of our experiments, we compared both U-Net versions for the best settings. It turned out that the light architecture showed better performance than the full architecture in terms of the PSNR (Figure A2a), while both architectures achieved the same FEL on the real image patch in terms of artifact level (Figure A2c). These results confirmed our choice of light architecture to find the best training settings.

For real-world images, we achieved the best performance when our lightweight U-Net-like architecture (10) was trained with the data augmented with ISO noise and varied exposure using the min-FEL criteria to select the best point in the parameter space. The choice of the FEL as an optimization criterion resulted in lowering FEL from 30.69% to 1.29%, which was visually perceived as the disappearance of artifacts in the real-world images.
